# Fucoidan and Derived Oligo-Fucoses: Structural Features with Relevance in Competitive Inhibition of Gastrointestinal Norovirus Binding

**DOI:** 10.3390/md19110591

**Published:** 2021-10-21

**Authors:** Franz-Georg Hanisch, Cem Aydogan, Horst Schroten

**Affiliations:** 1 Medical Faculty, Institute of Biochemistry II, University of Cologne, 50931 Köln, Germany; 2 PhytoNet AG, 8834 Schindellegi-Feusisberg, Switzerland; caydogan@phytonet.com; 3 Pediatric Infectious Diseases Unit, University Children’s Hospital Mannheim, 68167 Mannheim, Germany; horst.schroten@medma.uni-heidelberg.de

**Keywords:** fucoidan, norovirus, carbohydrate-lectin binding, food additive, mass spectrometry

## Abstract

Norovirus infections belong to the most common causes of human gastroenteritis worldwide and epidemic outbreaks are responsible for hundreds of thousands of deaths annually. In humans, noroviruses are known to bind to gastrointestinal epithelia via recognition of blood-group active mucin-type O-glycans. Considering the involvement of l-α-fucose residues in these glycans, their high valency on epithelial surfaces far surpasses the low affinity, though specific interactions of monovalent milk oligosaccharides. Based on these findings, we attempted to identify polyfucoses (fucans) with the capacity to block binding of the currently most prevalent norovirus strain GII.4 (Sydney, 2012, JX459908) to human and animal gastrointestinal mucins. We provide evidence that inhibitory effects on capsid binding are exerted in a competitive manner by α-fucosyl residues on *Fucus vesiculosus* fucoidan, but also on the galacto-fucan from *Undaria pinnatifida* and their oligo-fucose processing products. Insight into novel structural aspects of fucoidan and derived oligosaccharides from low-mass *Undaria pinnatifida* were revealed by GCMS and MALDI mass spectrometry. In targeting noroviral spread attenuation, this study provides first steps towards a prophylactic food additive that is produced from algal species.

## 1. Introduction

Noroviruses belong to the family of *Caliciviridae*, which are small non-enveloped viruses that contain a single-stranded RNA genome surrounded by a capsid protein. The capsid is composed of 180 copies of a major capsid protein, VP1, and small numbers of a minor capsid protein, VP2 [1]. Based on variations within the VP1 gene, noroviruses are classified into seven genogroups, termed GI-VII [2]. Strains of the genogroup GII are responsible for most infections in humans [3]. To induce an infection the norovirus needs to bind to the intestinal epithelia, a process that is at least partly mediated by specific interactions of a lectin-like activity in the norovirus capsid VP1 protein and blood group-active mucin-type O-glycans on membrane glycoproteins or mucins [4,5]. Accumulating evidence suggests that fucose, as part of histo-blood group antigens (HBGAs) or Lewis-like antigens, plays an essential role in the lectin-like recognition by the VP1 capsid protein of GII norovirus strains. This holds in particular true for the highly infectious GII.4 and the recently emerged GII.17 noroviruses. ABH- and Lewis fucoses of blood group antigens are introduced by the FUT2 and FUT3 fucosyltransferases and homozygously recessive individuals lacking these enzyme activities were less susceptible or even resistant to an infection by certain strains in human challenge and outbreak studies [6,7]. Certainly, in addition to blood group-related glycans, other host factors are required, as shown for feline calicivirus entry via junctional adhesion molecule 1 [8] and for murine norovirus entry via a novel cellular protein receptor [9].

Besides vaccine-based approaches to combat the virus, one of the alternative strategies to prevent norovirus infections is based on food additives, such as human milk oligosaccharides (HMOs). Human milk oligosaccharides represent an ideal source of potential competitors of viral glycan receptors, which mimic the structures of blood group-active mucin-type O-glycans. The trisaccharide 2-fucosyllactose (2′FL) is able to block norovirus binding quite efficiently [10,11], and has reached market approval as a safe food additive. We could in addition provide evidence for other milk oligosaccharides in the high-mass range that exert even stronger competitive effects on norovirus binding to gastric mucins [12]. During these studies we observed that oligo-valency of fucosyl residues in hepta- to decasaccharides provides a promotion of competitive effects on norovirus binding. This became most evident when l-fucose dendrimers with varying degrees of substitution and no relationship to blood group structures were compared with respect to their competitive activity [12]. High valency of terminally accessible α-l-fucose is a feature of natural polysaccharides belonging to the group of poly-fucoses or fucans. Algal fucoidans are present in several orders, mainly *Fucales*, and *Laminariales,* but also in *Chordariales*, *Dictyotales*, *Dictyosiphonales*, *Ectocarpales*, and *Scytosiphonales*. *Fucus vesiculosus* or bladderwrack belongs to the Fucales, whereas *Undaria pinnatifida* is a species of the Laminariales. Fucoidans exist either as a homopolymer of fucose, like in *Fucus vesiculosus* fucoidan (FvF) with their central chains composed of repeating (1 → 3) and (1 → 4) linked α-l-fucopyranose residues [13], or as a heteropolysaccharide, like in *Undaria pinnatifida* fucoidan (UpF), claimed to consist of Fuc1-3Gal1-3 repeats [14]. In all fucoidans sulfate residues are found at high densities, as every second monosaccharide can be substituted.

Fucoidans from brown algae have previously attracted much attention, as they were claimed to exert a series of health beneficial effects [13]. Like other sulfated polysaccharides, fucoidans can inhibit virus infection of cells. This has been demonstrated for *Herpes simplex*, cytomegalovirus, and human immunodeficiency virus [15] as well as bovine viral diarrhea virus [16], probably by competing with cell surface heparan sulfate for binding to the virus. Some data were provided that point to therapeutic applicability in the context of cytomegalovirus infection, but the observed effect was strictly dependent on the presence of sulfate on the polysaccharide.

We here report for the first time on a lectin-carbohydrate mediated, competitive anti-norovirus effect exerted by α-l-fucosyl residues of fucoidans and desulfated/fragmented processing products on viral capsid binding to gastrointestinal mucins. We were able to identify structural details of the polysaccharides from *Fucus vesiculosus* (FvF) and from the less characterized algal species *Undaria pinnatifida* (UpF) partly with relevance for the binding to noroviral capsids and in conflict with published data. The major point we tried to address in this study is the avidity of fucoidan binding to the noroviral capsids, which should depend mainly on the valencies of terminally accessible fucosyl residues and, hence, on the degree of polysaccharide branching. The two fucoidan species under study were chosen, as they exhibit distinct degrees of backbone branching. To generate low-mass fucoidans for structural studies by mass spectrometry, two approaches of fragmentation by mild acid hydrolysis with polystyrene sulfonic acid and by hydrothermal degradation were followed. While the former expectedly generates largely desulfated oligosaccharides, similar to the application of mild acid hydrolysis with dilute hydrochloric acid, the applicability of the latter approach is less well explored.

## 2. Results

### 2.1. Fucoidans Compete with Blood Group Fucosyl Residues on Gastric Mucins for Binding to Noroviral VLPs

**Figure 1:**
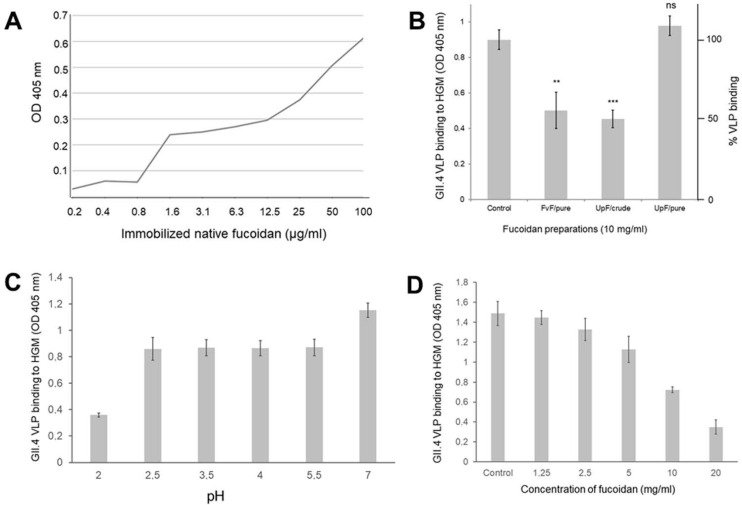
Fucose residues on fucoidans can serve as decoys in the blocking of noroviral VLP binding to human gastric mucins. (**A**) noroviral VLP binding to immobilized fucoidan from purified *Fucus vesiculosus* (duplicate assay); (**B**) pure and nutritional-grade (crude) fucoidan preparations from *Fucus vesiculosus* (FvF) and *Undaria pinnatifida* (UpF) exert distinct inhibitory effects on VLP binding to human gastric mucins (triplicate assay). Statistical significance of differences in VLP binding based on the student’s *t*-test is indicated as * *p* < 0.05, ** *p* < 0.01, *** *p* < 0.001 or as non-significant (ns). VLP binding in the presence of UpF/pure was not significantly different from controls (*p* value: 0.07); (**C**) GII.4 VLP binding to human gastric mucins at varying pH conditions (triplicate assay); (**D**) GII.4 VLP binding is blocked by FvF in a concentration-dependent manner (triplicate assay).

Initial assays were designed to demonstrate viral capsid binding to immobilized fucoidan from *Fucus vesiculosus* and were based on virus-like particles (VLPs) of the most endemic strain GII.4 (Sydney, 2012, JX459908) (Figure 1A). To evaluate applicability of fucoidans as a food additive with competitive inhibitory effect on noroviral binding to epithelial mucins, we established solid-phase binding assays on immobilized human gastric mucins. Fucoidans from different algaeal species (*Fucus vesiculosus*, *Undaria pinnatifida*) and fucoidan preparations with varying grades of purity (purified or nutritional grade) revealed inhibitory capacities of the native polysaccharides (Figure 1B). Purified FvF inhibiting about 50% of VLP binding at 10 mg/mL served as a reference to estimate inhibitory activities of UpF preparations. We could demonstrate that purified UpF was apparently inactive, whereas the crude, nutritional-grade preparation was slightly more active in VLP blocking compared to the FvF reference.

Binding to gastric mucins of VLPs from GII.4 (Sydney, 2012, JX459908) showed a characteristic dependency on the pH of buffers used in capsid binding assays (Figure 1C). As expected, the VLP binding decreased at lower pH to about 75%, remained constant at this level down to pH 2.5, and decreased to about 20% at pH 2.0.

The inhibitory effect of FvF on GII.4 VLP binding was concentration-dependent from 1 to 20 mg per ml (Figure 1D) indicating that it was competitive. An IC_50_ was determined for FvF at approx. 10 mg/mL corresponding to 262 µM for an average sized fucoidan in FvF preparations reported to be 38.2 kD [17].

### 2.2. Sulfate Residues on Fucoidan Are Not Essential for the Blocking of VLP Binding to Gastric Mucins

**Figure 2:**
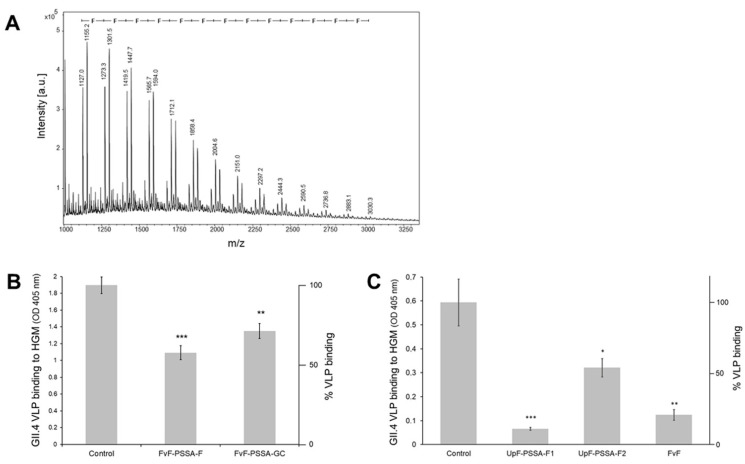
Desulfation/fragmentation processing of fucoidans by mild acid hydrolysis with polystyrene sulfonic acid and determination of VLP blocking activity profiles. (**A**) MALDI mass spectrometry in the reflectron, positive-ion mode of permethylated FvF-derived oligosaccharides; (**B**) blocking activity profile of PSSA-processed FvF (5 mg/mL) in the dialysis filtrate (cut-off 6–8 kD) and solid-phase extracted on graphitized carbon (GC); (**C**) blocking activity profile of PSSA-processed UpF in the dialysis filtrates F1 (neutralized before concentration) and F2 (neutralized after concentration), pure FvF served as positive control of blocking activity. (**B**,**C**) Statistical significance of differences in VLP binding based on the student’s *t*-test is indicated as * *p* < 0.05, ** *p* < 0.01, *** *p* < 0.001 or as non-significant (ns).

Polysulfonic acid-catalysed hydrolysis of FvF (refer to method A) resulted in the formation of partially desulfated low-molecular mass fucoidan oligosaccharides, as revealed by positive-ion MALDI mass spectrometry of native reaction products (Figure 2A). The sizes of oligo-fucoses ranged from 2- to 20-mers, the most abundant species were hepta- to decamers. The sulfate-free oligo-fucoses contained in the dialysis filtrate and in the eluate of carbograph solid-phase extraction columns were active as inhibitors of VLP binding to gastric mucins (Figure 2B). These findings strongly indicate that the observed inhibitory effect is independent of sulfation of the polyfucose and is merely based on the interaction of sulfate-free fucoses with the lectin-like viral capsid protein (see also [12] for the effect of sulfation).

Fucoidan from *Undaria pinnatifida* was processed in the same way to generate desulfated oligosaccharides and to test their VLP binding blocking efficiencies relative to the native polysaccharide. While the pure preparation had been shown to exhibit no measurable effects on the VLP binding to gastric mucins (Figure 1B), the processed UpF-derived oligosaccharides were strongly active (Figure 2C). Two charges of mild acid (PSSA)-treated, low-mass fractions were tested: F1and F2 neutralized before or after concentration by vacuum rotation. The weaker inhibitory activity of F2 may result from partial degradation of oligosaccharides during the drying process due to increasing concentrations of sulfuric acid liberated by hydrolytic desulfation.

### 2.3. Desulfated Fucoidan Oligosaccharides from FvF Reveal Unbranched Chains in the Size Range Up to Nonasaccharides

**Figure 3:**
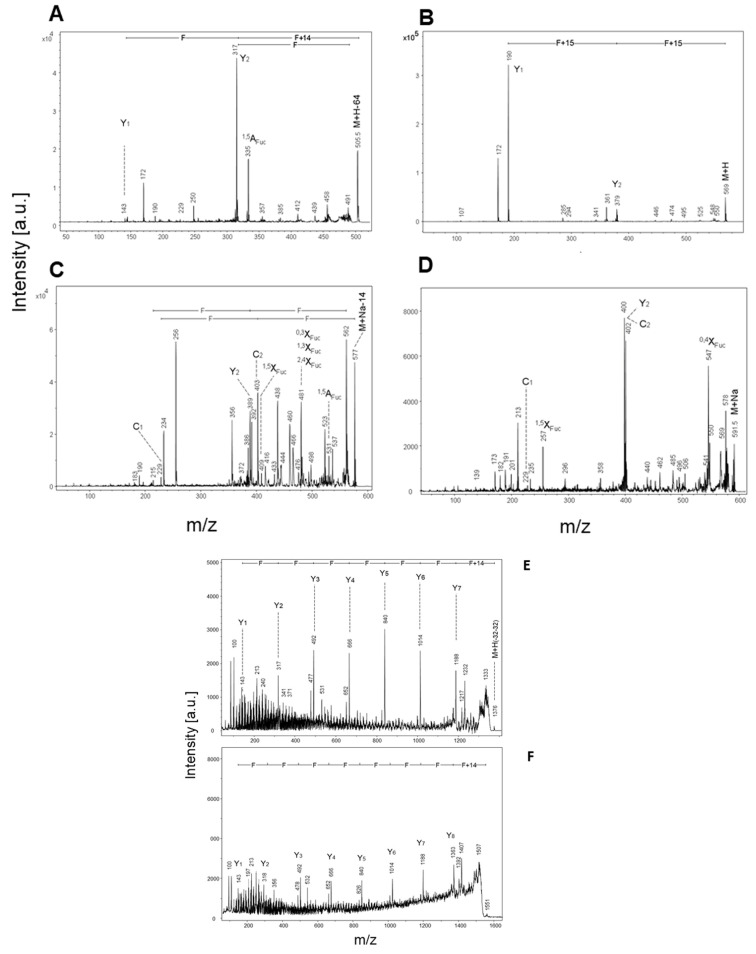
MALDI mass spectrometry in the post-source decay mode (MS2) of permethylated oligofucoses from FvF. (**A**) Fragment spectrum of a trifucose detected as M+H-64 at *m*/*z* 505; annotation of primary and secondary fragments and of internal or cross-ring fragments followed the nomenclature of Domon and Costello [19]; (**B**) Fragment spectrum of a trifucose detected as M+H at *m*/*z* 569; (**C**) Fragment spectrum of a trifucose detected as M+Na-14 at *m*/*z* 577; (**D**) Fragment spectrum of a trifucose detected as M+Na at *m*/*z* 591; (**E**) Fragment spectrum of a octafucose detected as M+H-64 at *m*/*z* 1376; (**F**) Fragment spectrum of a nonafucose detected as M+H-64 at *m*/*z* 1551.

As reported above, the application of mild acid hydrolysis (method A: polystyrene sulfonic acid, 60 °C) resulted in the apparently complete removal of sulfate residues with concomitant fragmentation to oligo-fucoses with sizes ranging up to about 20-mers. The underivatized oligosaccharides were detected in series of molecular ions corresponding to M+Na, M+Na-28, M+Na-56, and M+Na-84 pointing to cross-ring cleavages at the reducing sugar during ionization and in-source decay (Figure 2A). The permethylated oligosaccharides revealed series of molecular ions M+Na, M+Na-14, M+H, M+H-14, and M+H-64, indicating partial undermethylation at C1 of the reducing fucose and methanol elimination during ionization. Accordingly, for the trifucose a series of parent ions were detected at *m*/*z* 591, 577, 569, 555, and 505. Figure 3A–D shows MS/MS fragmentation spectra of these parent ions with dominant Y, Z and C-type ions and some internal and cross-ring fragments in accordance with the structural assignment of a trifucose Fuc1-3Fuc1-3Fuc. The same holds true for the MS/MS spectra measured for M+H(-32-32) parent ions detected at *m*/*z* 1375 and 1550 that correspond to octa- and nonafucoses (Figure 3E,F). The sulfate-free octa- and nonasaccharides yielded complete series of strong Y-type fragments in PSD-MALDI-MS indicating that the oligofucose chains in this size range did not contain branching points (refer to Y1 to Y7 in Figure 3E and to Y1 to Y8 in Figure 3F). The finding is at least partly in conflict with the revised structure model published by Patankar et al. [18], who claimed that fucoses linked (1–2) or (1–4) to the (1–3)-linked core form branch points, one for every 2–3 fucose residues within the chain.

### 2.4. Hydrothermal Degradation of Fucoidans

**Figure 4:**
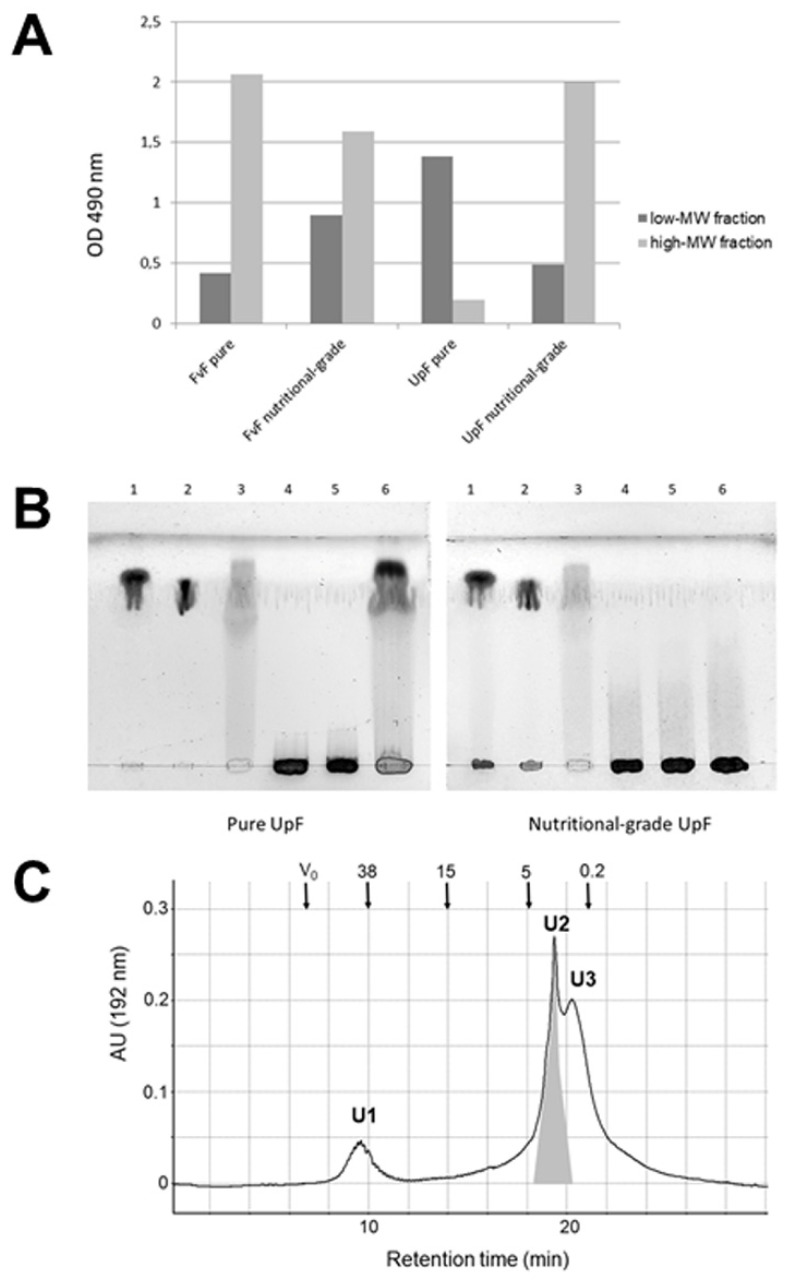
Hydrothermal degradation of pure and nutritional-grade fucoidans from *Fucus vesiculosus* and *Undaria pinnatifida.* (**A**) HTD followed by ultrafiltration of oligosaccharides over 10 kD cut-off membranes. Low-MW and high-MW fractions are marked as dark-grey or light-grey bars. Sugars were detected by the phenol-sulfuric acid assay. (**B**) Thin-layer chromatography of fractions obtained after HTD (120 °C) of pure or nutritional-grade UpF for 0, 30 min or 60 min. Samples were applied in lanes 1 (l-fucose), 2 (d-galactose) and 3 (oligofucoses prepared from FvF by mild acid hydrolysis), 4 (HTD 0 min), 5 (HTD 30 min), 6 (HTD 60 min). Sugars were detected by spraying with 10% sulfuric acid in ethanol and heating at 120 °C. (**C**) Low mass UpF was generated by HTD (120 °C, 120 min) and ultrafiltrated over 10 kD membranes before GPC by HPLC on Biosep SEC-S3000. Arrows indicate either the void volume (V_0_) or estimated molecular masses (kD) according to calibration standards derived from FvF.

To generate low-molecular mass fucoidan from FvF or UpF under milder, acid-free conditions we chose the application of hydrothermal degradation. Treatment of a concentrated solution of different fucoidan preparations at 120 °C for 120 min revealed varying fragmentation efficiencies dependent on the sources and grades of purity of fucoidans. Most obvious was that FvF (purified or nutritional grade) showed higher stability under these conditions, as only fractions of the polysaccharide were degraded and able to pass ultrafiltration membranes with a cut-off of 10 kD (Figure 4A). On the other hand, purified UpF was degraded much more efficiently to low molecular mass fucoidan (LMF) than the nutritional-grade UpF indicating that chemical treatments during the purification process had structurally modified the polysaccharide. Purified FvF (Marinova) should have a lower MW than the nutritional-grade charges due to partial degradation during treatment with alkali. Despite this, most of the purified FvF remains in the high-MW ultrafiltrate with molecular masses above 10 kD. On the other hand, UpF purified by Marinova in the same way is found mostly in the LMF fraction after hydrothermal degradation. This holds not true for the nutritional-grade material, which emphasizes that the purification process pre-degrades and modifies the UpF polysaccharide potentially by removal of O-acetylation.

By reducing the reaction times of hydrothermal degradation, we could demonstrate that the fragmentation of purified UpF was extensive already after 60 min. On the other hand, the nutritional-grade quality remained stable under these conditions, as revealed by thin-layer chromatography (Figure 4B). While most of the processing products comigrate with the mono- to disaccharides, the presence of larger oligosaccharides is indicated by smears over a broader range of oligosaccharide sizes.

The ultrafiltrated fraction of UpF after 2 h of hydrothermal degradation (LMF) was separated by gel-permeation chromatography on Biosep SEC-S3000 (Figure 4C). The UV profile registered at 192 nm yielded two major subfractions U2 and U3. The latter fraction was isographic with smaller oligosaccharides in the range of mono- to trisaccharides. Fraction U2 was further analyzed by MALDI-MS and GC-MS of the methylated oligosaccharides.

### 2.5. Structural Data on UpF and Its Hydrothermal Degradation Products

**Figure 5:**
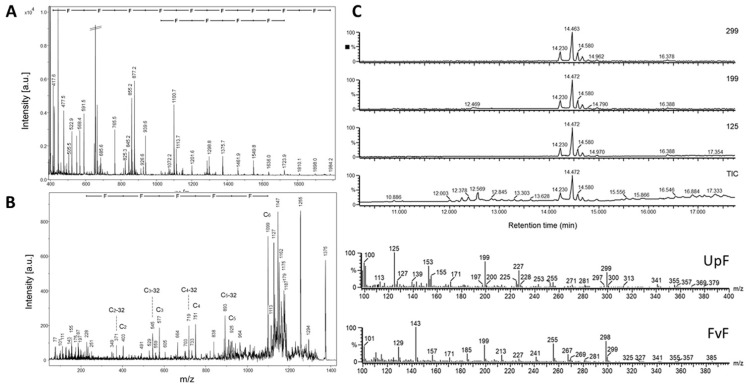
Hydrothermal degradation of pure UpF results in the liberation of linear oligofucoses. (**A**) positive-ion MALDI mass spectrum of permethylated oligofucoses (up to dp11) obtained by HTD (120 °C, 120 min); (**B**) PSD-MALDI mass spectrum of parent ion M+H-64 at *m*/*z* 1375; annotation of fragment ion masses followed the nomenclature of Domon and Costello [19]; (**C**) gas chromatography-mass spectrometry of methylated oligosaccharides obtained by HTD of UpF. The upper panel shows chromatographic traces by recording the total ion current (TIC) or specific fragment masses at *m*/*z* 125, 199, or 299. The lower panel shows electron impact mass spectra recorded at 70 eV for the oligosaccharide eluting at 14.47 min (UpF) and for a trifucose derived from FvF by partial acid hydrolysis with 10 mM hydrochloric acid at 60 °C for 4h.

Most significantly, data on oligosaccharides in fraction U2 revealed by GC-MS were in conflict with established structure models of UpF. In the literature UpF is claimed to be composed of hetero-disaccharide repeats of the structure Fuc1-3Gal1-3 [14]. However, oligosaccharides in fraction U2 detectable as proton adducts and methanol elimination products (M+H-32-32) could be identified as oligo-fucoses (Figure 5A). Oligosaccharides up to undecasaccharides were detectable in the LMF fraction. PSD-fragmentation analysis for the octasaccharide MH-64 at *m*/*z* 1375 revealed a continuous series of C-type fragments indicating a non-branched core structure of the oligofucoses (Figure 5B). Trifucoses eluting in the GC eluant at 14–15 min were characterized in electron impact mass spectra by the formation of dominant primary ions at *m*/*z* 125, and *m*/*z* 299 (Figure 5C). Interestingly, similar fragment series were obtained for oligosaccharides derived by mild acid desulfation-fragmentation (method B) from FvF, which is known to be composed exclusively of fucoses. Referring to a trifucose (Fuc1-3Fuc1-3Fuc), the respective primary fragments can be assigned as Z_1_ (*m*/*z* 125) or Z_2_ ions (*m*/*z* 299), together with several cross-ring fragment ions, like ^1,4^X_Fuc_ or ^0,2^X_Fuc_ (*m*/*z* 199) and ^1,5^A_Fuc_ (*m*/*z* 335), or ions formed by double fragmentation, like ^0,3^X_Fuc_Y (*m*/*z* 229).

**Figure 6:**
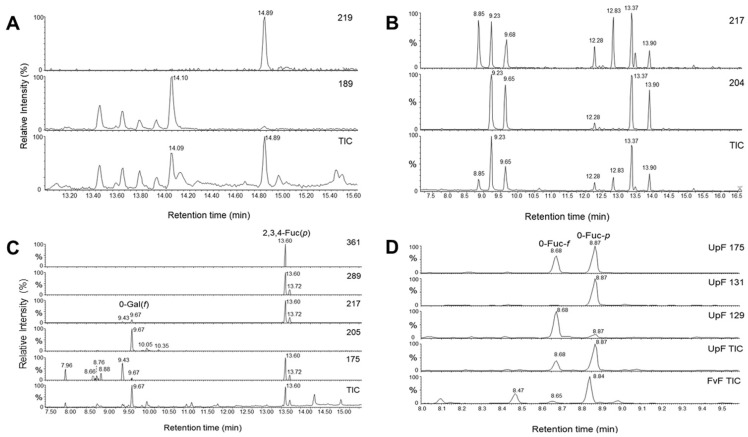
Structural characterization of oligosaccharides derived from pure UpF by HTD. (**A**) gas chromatography-mass spectrometry of methylated oligosaccharides revealing non-reducing hexose (peak at 14.89 min in the *m*/*z* 219 ion trace) or non-reducing desoxyhexose (peak at 14.10 min in the *m*/*z* 189 ion trace). (**B**) monosaccharide composition data of UpF (pure) determined after methanolysis by GCMS of trimethylsilylated 1-O-methylglycosides. The peaks correspond to furanosidic l-fucose (8.85 min) or pyranosidic α (9.23 min) or β-l-fucose (9.68 min), or to furanosidic d-galactose (12.83 min), or the pyranosidic α- (13.37 min) or β-d-galactoses (13.90 min). (**C**) methylation-linkage analysis by GCMS of methylated oligosaccharides in fraction U2 from GPC of HTD-processed UpF (pure). The chromatographic profiles of the TIC are shown together with selected fragment ion masses specific for terminal desoxyhexose (*m*/*z* 175), hexose (*m*/*z* 205), or triply substituted (desoxy)hexoses (*m*/*z* 217, 289, 361). The identity of major PMAA representing oligosaccharides in fraction U2 were annotated. (**D**) The TIC profiles of PMAA from U2 were enlarged for the retention time range between 8.0 and 10.0 min to focus on the proportions of furanosidic vs. pyranosidic l-fucose. Compared to PMAA profiles of oligosaccharides from FvF, which had been prepared by mild acid desulfation/fragmentation, the terminal furanosidic l-fucose (0-Fuc-*f*) relative to its pyranosidic counterpart (0-Fuc-*p*) represents about 30% (compared to less than 10% in the FvF preparation). Figures (**A**–**D**): chromatographic profiles were normalized according to the most intense signal at 100% (y-axes giving throughout the relative intensities in %).

In agreement with published data on UpF, the monosaccharide composition in the nutritional-grade preparation is dominated by nearly equimolar contents of l-fucose and d-galactose (Figure 6A). Although the purified UpF sample revealed slightly higher relative contents of l-fucose (Table 1), the ratio is still near 1/1 and agrees with the claimed heteromeric core unit Fuc1-3Gal1-3. After hydrothermal degradation however, a striking shift to about two-fold higher fucose contents is observed in the ultrafiltrated, low mass fraction (Figure 6A and Table 1). This finding is not in accordance with the published structural model of UpF, as it indicates a selective degradation of a fucan in mixture or in structural unit with a galactan.

To estimate the average degrees of polymerization (dp) of low mass UpF fractions obtained by either mild acid hydrolysis with PSSA or by HTD, the reducing termini of oligo-/polysaccharides were converted to the respective alditols (fucitol, galactitol) prior to monosaccharide analyses (Table 2). In this way, the relative contents of Fuc/Fuc-ol or Gal/Gal-ol reveal quantitative estimations of the average chain lengths of the polymers (Figure 6B). While the native UpF sample contained only trace amounts of alditols, the chromatograms of processed samples containing oligosaccharides of varying lengths revealed significant amounts of reduced sugars with Fuc-ol/Gal-ol ratios exceeding 10 or even 100. This finding could be interpreted by the assumption that fucosyl residues were cleaved at faster rates than galactosyl residues during mild hydrolysis or hydrothermal degradation. Referring to the ratios of fucose and its alditol (Fuc/Fuc-ol) the average chain lengths of oligosaccharides generated by mild acid hydrolysis (PSSA) were about fourfold higher than those after HTD. It is obvious that samples processed by HTD contain mostly mono- and disaccharides and that oligosaccharides of larger chain lengths represent a minor subfraction. These estimations are in agreement with the GPC profile, where the bulk of processing products comigrated with the mono- to disaccharides (Figure 5C).

One distinct subfraction (U2) could be separated by GPC from the bulk of mono- and disaccharides (fraction U3 in Figure 5C), which revealed distinct structural features in linkage analyses (Figure 6C). U2 oligosaccharides are mainly characterized by terminal furanosidic galactose (peak at 9.7 min and primary fragment ions at *m*/*z* 118, 161, 205) and tri-substituted fucose (peak at 13.6 min and fragment ions at *m*/*z* 145, 217, and 289). A further structural feature of potential significance can be seen in the much higher proportion of furanosidic vs. pyranosidic l-fucose, when comparing UpF with FvF oligosaccharides (Figure 6D).

## 3. Discussion

### 3.1. Fucose Multi-Valency as an Approach for Norovirus Drug Design

Our previous work had identified a series of HMOs with binding inhibitory potential: 2′FL, 3FL, LNFP-I, LNneoFP-I and oligo-fucosylated octa- to decasaccharides as possible broadly reactive anti-norovirals. However, monofucosylated species, like blood-group H1-active lacto-*N*-fucopentaose I, revealed strikingly low affinities in competition experiments with multivalent receptors on the viral capsids. We concluded that multivalency of fucose might be the more important feature compared with specificity for a particular blood group glycan. For this reason, we designed blood group-unrelated oligo-fucose dendrimers with higher competitive capacities, and demonstrated strongly increased capsid blocking effects. As an alternative to synthetic compounds, like oligo-fucosylated cyclodextrine (FCD) dendrimers, natural polysaccharides were evaluated as anti-noroviral agents [12].

In extension of previous work, we here explored polyfucoses or fucans and their potential to block viral capsid binding to mucins, either as native fucoidan or as processing products derived from the brown algal polysaccharides by partial acid hydrolysis or hydrothermal degradation. Although these oligo-l-α-fucoses in the size range of 2–20 dp do not exhibit any structural relationship to blood group glycans, they revealed strong inhibitory potentials in norovirus binding to human gastric mucins. The inhibitory effect was shown to be concentration-dependent, but independent of the sulfate content of the polysaccharide or its processing products [12], an observation which is clearly in conflict with the established relationship between sulfate groups and various biological activities of fucans [20]. Both findings point to a competitive effect mediated by multiple unmodified fucose residues of fucoidan in interaction with the VP1 capsid protein. A direct, but comparatively weak interaction with single fucose residues was revealed in X-ray crystallography of oligofucose-P domain co-crystals (G.H. Hansman, personal communication). The findings are in accordance with results from a recent study based on fucose-functionalized precision glyco- macromolecules for targeting the human norovirus capsid protein [21].

Two recent reports support the antiviral effects of fucoidans by demonstrating inhibitory effects of *Undaria pinnatifida* fucoidans against murine noroviruses in vitro [22] or of *Fucus vesiculosus* fucoidans against human noroviruses in zebrafish larvae in vivo [23]. However, the reported findings seem to have a totally distinct mechanistic background, as the observed effects are dependent on cell entry and are hence exerted intracellularly. Moreover, the inactivity of 2′-fucosyllactose in the in vivo model is in striking conflict with the inhibitory potential of this and other fucosylated milk oligosaccharides and of desulfated fucoidan oligosaccharides that exert competitive effects on noroviral binding to epithelial mucins [12]. Discriminating the mechanisms which underly the reported fucoidan effects on noroviral infection or cellular replication, it is obvious that sulfation of fucoidans is not involved in binding inhibition, but certainly plays a role in prevention of cellular replication of the virus.

### 3.2. Essential Structural Features of UpF

To understand the essential structural features of fucoidans under study with respect to their activities in noroviral blocking efficiencies, we attempted to fragment the polysaccharides to the level of oligosaccharides by mild acid hydrolysis (FvF, UpF) or hydrothermal degradation (UpF). Mass spectrometric analysis of the methylated oligosaccharides revealed several unexpected structural features for both fucoidans under study. FvF is known to be mostly composed of l-fucose, reflected in an oligofucose series with up to dp 20. On the other hand, it is striking to find similar series of oligofucoses in the hydrothermal degradation of UpF, which is claimed to be composed of alternating l-fucose and d-galactose in Fuc1-3Gal1-3 repeats [14]. On comparison of their overall structural features, FvF has reportedly a more branched backbone with branching points at every second to third monomer, whereas UpF had been shown be less branched [24]. Deviating from this report, we found similar core structures for both fucoidans with unbranched oligofucose chains up to octa- or nonasaccharides. Strikingly, the pure fucoidans (UpF, FvF) exhibit different efficacies in the blocking of noroviral capsid binding to gastrointestinal mucins. While pure FvF is inhibitory active, the respective UpF charges did not show measurable effects (Figure 1B). Contrasting with this, the nutritional-grade charges of UpF were similarly active in VLP binding inhibition. These findings are difficult to explain solely on the basis of backbone branching and varying numbers of exposed fucosyl residues, although multivalency plays expectedly a major role. Oligofucoses in processed low mass fucoidans are generally of slightly higher inhibitory activity than the native polysaccharides. However, the effect of depolymerization and associated increase in exposed/terminal fucoses is opposed by the decreasing valencies of smaller fragments.

There are increasing numbers of reports on biological effects exerted by fucoidans from various species [25]. Among others they were claimed to be involved in the control of acute and chronic inflammation and to have potentially therapeutic pleiotropic anti-inflammatory effects. Of considerable importance in this context is the inhibition of complement binding to C1q receptors present on endothelial cells, since it was shown that branching of fucoidan oligosaccharides is a major factor in this activity at the conformational level [26]. These findings are of considerable interest, as they could explain some of the discrepancies between structural features of FvF vs. UpF and their biological activities.

That spatial arrangements of exposed fucoses could play a role was shown for the noroviral strain GII.10, where the protruding P domains on the capsid surface exhibit additional binding sites for l-fucose [21]. Hence, steric aspects could contribute tremendously to the multivalent interaction with fucose ligands on fucoidan as entry inhibitors of norovirus infections.

In conclusion, conformational effects could come into play and modulate the strength of competitive fucoidan interaction with noroviral capsids. Not merely the valencies of terminally accessible fucose residues, but also their regio-spatial, arrangements should have impact on the avidity of fucoidan inhibition of noroviral entry. Further studies by NMR will certainly contribute to a better understanding of the steric prerequisites for the cooperative multivalent interaction between the noroviral lectin and fucoidan oligosaccharides and support the design of effective food additives as inhibitors of viral entry and infection.

## 4. Experimental Procedures

### 4.1. Materials

Fucoidan isolated from *Fucus vesiculosus* was purchased from Sigma-Aldrich, Munich, Germany (Sigma F-5631, crude fucoidan from *Fucus vesiculosus*, and Sigma F-8190), or provided by Marinova PTY Ltd., Cambridge TAS, Australia (pure and nutritional-grade fucoidans from *Fucus vesiculosus*, and pure and nutritional-grade fucoidans from *Undaria pinnatifida*, UpF). Nutritional-grade UpF and FvF were used which both are certified by Generally Recognised as Safe (GRAS), USA, EU Novel Foods approval, TGA Listed Ingredient, Australia, Compliant with Algae Product requirements, China, and KFDA registered, Korea.

### 4.2. Processing of Fucoidan by Partial Acid Hydrolysis with Polystyrenesulfonic Acid

A total of 30 mg of fucoidan was solubilized at 10 mg/mL in water containing 8.4 mg polystyrenesulfonic acid (0.04 mM) and filled into a dialysis bag (cutoff: 6–8 kD) that was placed in 50 mL water and heated to 60 °C. The filtrate was continuously pumped over 580 mg graphitized carbon (24 mL/h) during 16 h. After washing with water (10 mL), the bound oligofucoses were eluted with 2 mL fractions of 30% ethanol in water. Oligofucoses passing the solid-phase extraction column and remaining in the 50 mL filtrate made up approx. 7.5 mg, oligofucoses bound to the column were estimated to range at 3 mg. Accordingly, most of the processing products did not bind effectively to graphitized carbon during inline application. Alternatively, a 10 mL fraction of the filtrate was applied offline onto a carbograph cartridge as described below.

### 4.3. Fractionation of Fucoidan Processing Products and Alternative Workup Strategies

Desalting of the processing products was performed by solid-phase extraction on carbograph cartridges (150 mg graphitized carbon, Grace). The cartridge was activated with 2 mL 80% acetonitrile in 0.1% aqueous TFA and equilibrated with 3 mL of water. After sample application in water, the column was washed with 2 mL water and oligosaccharides were eluted with 30% ethanol in water. A 150 mg carbograph column has the capacity to bind up to 40 mg hexoses; however, oligo-fucoses derived from fucoidan bound much less effectively (about 5 mg).

### 4.4. Hydrothermal Degradation of Fucoidans

To achieve partial fragmentation of fucoidans without affection of sulfation, nutritional-grade and purified samples of FvF and UpF were processed by hydrothermal degradation [27] as 20 mg/mL water solutions in screw-capped glass vials for 30 min to 120 min at 120 °C (not microwave-assisted). After cooling to room temperature, the reaction mixture was ultrafiltrated in 0.5 mL Amicon centrifugation devices (cutoff 10 kd). Sugars in 50 µL solutions were detected in duplicate assays by adding equal volumes of phenol (5%) and 250 µL sulfuric acid. Absorbances were determined after 30 min at 490 nm. The LMF fraction was chromatographed by size-exclusion chromatography on Biosep SEC-S3000 (300 × 7 mm, Phenomenex, Aschaffenburg, Germany) and oligosaccharides were isocratically eluted at a flow rate of 0.5 mL/min and detected at 192 nm. A further aliquot of the LMF fraction were withdrawn and applied onto HPTLC silica plates to separate the products in a solvent system of acetic acid, ethanol, and water (40/40/20, *v*/*v*/*v*) and to detect the sugars after spraying with 10% sulfuric acid in ethanol by heating for 5 min at 120 °C. Another aliquot of the LMF fraction was dried and methylated for analysis by GCMS (see above).

### 4.5. Production of Norovirus VLPs

Three different strains of the genogroup GII were included in this study: GII.4 (Sydney, 2012, JX459908), GII.17 (Kawasaki308, LC037415) and GII.10 (Vietnam 026, AF504671). VP1 protein was recombinantly expressed in insect Sf9 cells as previously described [28]. Briefly, Sf9 cells were transfected with recombinant VP1 bacmids using Effectene. The cells were incubated at 26 °C and harvested 6 days postinfection. The baculovirus harvest was clarified by low-speed centrifugation and was used to infect Tn5 cells at 26 °C. The cells were then harvested 6 days postinfection. The VLPs were concentrated by ultracentrifugation and then purified by CsCl equilibrium gradient ultracentrifugation. The VLP band was collected from the side of the tube and the VLP morphology was examined using electron microscopy.

### 4.6. Norovirus Capsid Binding and Binding Inhibition Assays

VLP binding and inhibition assays were performed on immobilized human gastric mucins (HGM, 10 µg/mL) from pooled gastric juice of blood group undefined subjects. Active polystyrene plate surface was blocked with 5% BSA/PBS for 1 h at 37 °C before VLPs were applied at 10 µg protein per ml of 0.05% Tween 20/PBS. After incubation for 1 h at 37 °C and threefold repeated plate washing with 0.5% BSA/PBS bound VLPs were detected with polyclonal rabbit antiserum to GII.4 VP1 capsid protein (diluted 1:3000 in 0.5% BSA/PBS). Anti-rabbit-Ig-alkaline phosphatase (AP), diluted 1:5000 in 0.5% BSA/PBS (GII.4), was used for development (1 h at 37 °C), followed by three washing steps and addition of the substrate *p*-nitrophenylphosphate (1 mg/mL) in 0.1M diethanolamine buffer, pH 9.8, containing MgCl_2_ (0.5 mM). Plates were read at 405 nm in a Tecan Sunrise (Remote Control) run with Magellan software.

Pretesting of VLP-fucoidan binding was performed on polylysine-pre-coated polystyrene microtitration plates by loading serial dilutions of FvF in concentrations up to 0.1 mg/mL to the wells (duplicate assays). VLP binding and immune detection was performed as described above.

In binding inhibition experiments, appropriate amounts of inhibitor were dried from stock solutions and resolubilized in 0.5% Tween 20/PBS containing 10 µg protein/mL VLP suspension. After 15 min preincubation the suspension of VLPs was applied onto the polystyrene-immobilized human gastric mucin (10 µg/mL) in 50 µL aliquots per well. Immunochemical detection of bound VLPs and plate development was performed as described above. Binding inhibition tests were performed in triplicate throughout. Statistical analyses were performed by applying the student’s *t*-test on the triplicate VLP binding assays. Significant differences were considered when *p* was <0.05.

### 4.7. Structural Studies by MALDI Mass Spectrometry

Underivatized oligofucoses derived from fucoidan were analysed by MALDI MS and MS/MS on a Bruker Ultraflextreme TOF/TOF instrument (Bruker, Bremen, Germany) in the reflectron positive-ion mode. The native glycans were applied onto stainless steel targets by mixing 0.5 µL aliquots solubilized in water with 1 µL aliquots of the matrix 2,5-dihydroxy benzoic acid in acetonitril/0.1% aqueous trifluoroacetic acid (1:2, *v*/*v*). Permethylated glycans were applied as methanol solutions by mixing of 0.75 µL with an equal volume of saturated 4-hydroxy-α-cinnaminic acid in 50% acetonitrile/0.1% aqueous trifluoroacetic acid.

### 4.8. Linkage Analysis by GCMS

Permethylation of glycans was performed as previously described [29]. The completeness of derivatization was controlled by MALDI-MS and the oligosaccharides were analysed by GCMS or were hydrolysed, reduced and acetylated to obtain partially methylated alditol acetates (PMAA) prior to GCMS analysis. Analyses were performed on a Fison MD800 mass detector (Fison/Thermo) coupled to a gas chromatograph (GC8000 series). The gas-chromatographic separation of oligosaccharides up to trisaccharides and of PMAA was performed on a Restec Rxi-Sil MS capillary column (15m, 0.25 mm ID, 0.25 µm) using a temperature-gradient from 100–300 °C with 6 °C/min (methylated oligosaccharides) or from 60–100 °C with 40 °C/min, and 100–260 °C with 10 °C/min (PMAA).

## Figures and Tables

**Table 1:** Relative contents of monosaccharides l-fucose and d-galactose in *Undaria pinnatifida* fucoidan and its processing products.

Sample *	Molar ratio l-Fuc/d-Gal	Estimated Molecular Masses (kD)
UpF (nutritional-grade)	0.953	n.d.
UpF (pure)	1.226	n.d.
UpF-HTD	1.964	<10
UpF-PSSA	0.876	<6–8

^*^ Nutritional-grade and purified fucoidan from *Undaria pinnatifida* was provided by Marinova without specification of molecular masses of polysaccharides. UpF-HTD refers to hydrothermally degraded low mass fucoidans from *Undaria pinnatifida* prepared from UpF (pure) and fractionated by ultrafiltration (molecular mass cutoff: 10 kD). UpF-PSSA refers to low mass material generated by mild polystyrenesulfonic acid-catalyzed hydrolysis (molecular mass cutoff of dialysis membrane: 6–8 kD).

**Table 2:** Ratios of fucitol vs. galactitol and of fucose vs. fucitol in processed UpF samples after reduction and methanolysis.

Sample	Fuc-ol/Gal-ol	Fuc/Fuc-ol
UpF native	-	-
UpF PSSA filtrate	>100	0.78
UpF PSSA CG	>10	0.92
UpF HTD-1	>30	0.23
UpF HTD-2	>10	0.18

PSSA filtrate refers to the oligosaccharide fraction after passage of a dialysis membrane with a 6–8 kD cutoff, PSSA CG to the respective fraction after solid-phase extraction on graphitized carbon. HTD-1 and -2 refer to two independent preparations of UpF by hydrothermal degradation at 120 °C followed by fractionation on ultrafiltration devices (molecular mass cutoff: 10 kD).

## Data Availability

Raw files of mass spectrometric data will be made available on the open public platform GlycoPOST.

## Abbreviations

FvF: *Fucus vesiculosus* fucoidan; GCMS, gas chromatography mass spectrometry; HTD, hydrothermal degradation; MALDI-MS, matrix-assisted laser-induced ionization mass spectrometry; PSD, Post-Source Decay; PSSA, polystyrene sulfonic acid; UpF, *Undaria pinnatifida* fucoidan; VLP, virus-like particle.
